# Multifunctional thermosensitive hydrogel for synergistic antibacterial, anti-inflammatory and osteogenic effects to promote periodontal regeneration in periodontitis treatment

**DOI:** 10.1093/rb/rbag098

**Published:** 2026-05-21

**Authors:** Long Wang, Mingxi Wang, Runzi Zhang, Wanmeng Wang, Jin Wu, Chunbo Tang

**Affiliations:** Department of Dental Implantology, The Affiliated Stomatological Hospital of Nanjing Medical University, Nanjing 210029, China; State Key Laboratory Cultivation Base of Research, Prevention and Treatment for Oral Diseases, Nanjing Medical University, Nanjing 210029, China; Jiangsu Province Engineering Research Center of Stomatological Translational Medicine, Nanjing Medical University, Nanjing 210029, China; Department of Dental Implantology, The Affiliated Stomatological Hospital of Nanjing Medical University, Nanjing 210029, China; State Key Laboratory Cultivation Base of Research, Prevention and Treatment for Oral Diseases, Nanjing Medical University, Nanjing 210029, China; Jiangsu Province Engineering Research Center of Stomatological Translational Medicine, Nanjing Medical University, Nanjing 210029, China; Department of Dental Implantology, The Affiliated Stomatological Hospital of Nanjing Medical University, Nanjing 210029, China; State Key Laboratory Cultivation Base of Research, Prevention and Treatment for Oral Diseases, Nanjing Medical University, Nanjing 210029, China; Jiangsu Province Engineering Research Center of Stomatological Translational Medicine, Nanjing Medical University, Nanjing 210029, China; Department of Dental Implantology, The Affiliated Stomatological Hospital of Nanjing Medical University, Nanjing 210029, China; State Key Laboratory Cultivation Base of Research, Prevention and Treatment for Oral Diseases, Nanjing Medical University, Nanjing 210029, China; Jiangsu Province Engineering Research Center of Stomatological Translational Medicine, Nanjing Medical University, Nanjing 210029, China; Department of Dental Implantology, The Affiliated Stomatological Hospital of Nanjing Medical University, Nanjing 210029, China; State Key Laboratory Cultivation Base of Research, Prevention and Treatment for Oral Diseases, Nanjing Medical University, Nanjing 210029, China; Jiangsu Province Engineering Research Center of Stomatological Translational Medicine, Nanjing Medical University, Nanjing 210029, China; Department of Dental Implantology, The Affiliated Stomatological Hospital of Nanjing Medical University, Nanjing 210029, China; State Key Laboratory Cultivation Base of Research, Prevention and Treatment for Oral Diseases, Nanjing Medical University, Nanjing 210029, China; Jiangsu Province Engineering Research Center of Stomatological Translational Medicine, Nanjing Medical University, Nanjing 210029, China

**Keywords:** magnesium peroxide, polydopamine, thermosensitive hydrogel, antibacterial, anti-inflammatory, osteogenesis, periodontal regeneration

## Abstract

Periodontal disease therapy faces complex pathological challenges, including persistent anaerobic bacterial infection, excessive inflammatory activation and impaired alveolar bone regeneration. Monofunctional biomaterials thus fail to meet comprehensive clinical needs. Herein, we developed a multifunctional thermosensitive hydrogel delivery system. By encapsulating polydopamine-coated magnesium peroxide (MgO_2_@PDA) nanocomposites within a Pluronic F127-sodium alginate (F127-SA) matrix, the system achieves integrated synergistic therapy targeting the ‘antibacterial-anti-inflammatory-osteogenic’ axis. Systematic characterization demonstrated that the hydrogel exhibits rapid thermosensitive gelation and excellent shear-thinning properties. *In vitro* experiments confirmed that MgO_2_@PDA enables the sustained release of oxygen (O_2_) and magnesium ions (Mg^2+^), effectively inhibiting the proliferation of key periodontal pathogens, including *Porphyromonas gingivalis* and *Fusobacterium nucleatum*. Furthermore, the system downregulates the secretion of pro-inflammatory cytokines by macrophages stimulated with lipopolysaccharide (LPS). Notably, it significantly enhances osteoblast migration, differentiation and mineralization *in vitro*. In a rat periodontitis model, this hydrogel effectively attenuated local inflammatory responses, inhibited anaerobic pathogen proliferation and upregulated the expression of bone regeneration markers. In summary, this hydrogel integrates sustained drug delivery, microenvironment modulation and regenerative guidance functions, providing a novel strategy with clinical translation potential for the comprehensive treatment of periodontitis and periodontal tissue regeneration.

## Graphical Abstract

**Graphical Abstract:**
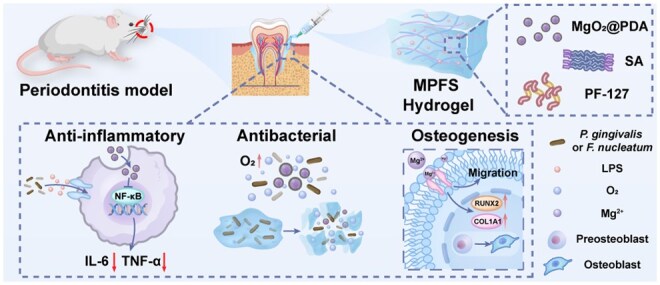

## Introduction

Periodontitis is a chronic, multifactorial inflammatory disease initiated by dental plaque accumulation, characterized by the progressive destruction of periodontal supporting structures (the periodontal ligament and alveolar bone). Its clinical manifestations include gingival bleeding, periodontal pocket formation and eventual tooth loss [1, 2]. Current therapeutic approaches, including supragingival scaling, subgingival root planning and surgical interventions, aim to eliminate biofilms and calculus. However, the complex anatomical architecture of periodontal pockets poses a major barrier to the deep penetration of therapeutic instruments, resulting in incomplete clearance of subgingival plaque and calculus, persistent gingival inflammation and progressive destruction of periodontal supporting tissues. This ultimately results in suboptimal treatment outcomes and high recurrence rates [3, 4]. Traditional local delivery systems (e.g. fibers [5], films [6] and microparticles [7]) rely predominantly on antibiotics, which exacerbate the escalating global crisis of antimicrobial resistance [8]. Meanwhile, monofunctional biomaterials are unable to address the multidimensionally intertwined pathological microenvironment of periodontitis. Constrained by their unimodal therapeutic profiles, they fail to simultaneously exert anti-inflammatory and osteogenic effects, let alone comprehensively target the interconnected pathological cascades underlying the disease. Thus, the development of a multifunctional, long-acting local delivery system integrating antibacterial, anti-inflammatory and regenerative properties has become an urgent unmet clinical need in periodontitis treatment [9].

Beyond bacterial colonization, the complex pathogenesis of periodontitis further underscores the need for multifaceted interventions, as it is intrinsically linked to an overactive host immune-inflammatory response [10]. Within this pathological microenvironment, this dysregulated immune response activates canonical inflammatory signaling pathways (e.g. nuclear factor κB, NF-κB), which drive osteoclast differentiation and bone resorption while suppressing osteoblast-mediated reparative functions [11], thereby exacerbating alveolar bone loss. Compounding this, local hypoxia—driven by dense inflammatory cell infiltration and impaired periodontal microcirculation—represents another key pathological feature [12]. This hypoxic niche stabilizes hypoxia-inducible factor-1α (HIF-1α), which amplifies inflammatory responses, promotes the survival of anaerobic pathogens and directly inhibits osteogenic activity [13]. Bacterial infection, excessive inflammation and local hypoxia thus form an intertwined vicious cycle that perpetuates tissue destruction, highlighting that an ideal therapeutic strategy must go beyond mere antimicrobial action to simultaneously modulate this deleterious inflammatory-hypoxic microenvironment, laying a solid foundation for effective tissue self-repair and functional regeneration.

To address the intertwined pathological hallmarks of hypoxia and inflammation-mediated tissue damage in the periodontal microenvironment, oxygen-releasing biomaterials capable of *in situ* oxygen generation have demonstrated significant therapeutic potential in recent years [14–16]. Among conventional oxygen donors, calcium peroxide (CaO_2_) is widely used but has a critical limitation: its decomposition drastically increases local tissue pH, creating an alkaline microenvironment that impairs the viability and osteogenic capacity of bone-regenerating cells [17]. In contrast, magnesium peroxide (MgO_2_) exhibits a pathology-responsive release profile: it reacts slowly with water, and its decomposition is accelerated in acidic microenvironments, facilitating the sustained and targeted release of oxygen (O_2_) and magnesium ions (Mg^2+^) [18]. Notably, Mg^2+^ is not a passive byproduct but a bioactive molecule with multifaceted pro-regenerative properties: it modulates the early inflammatory microenvironment to direct monocyte-macrophage recruitment and polarization toward a reparative phenotype, stimulates the osteogenic differentiation of stem cells and enhances endothelial cell migration [19, 20], collectively disrupting the ‘inflammation-bone loss’ vicious cycle. Recent studies have explored the application of MgO_2_ in antibacterial therapy and bone tissue regeneration, confirming its feasibility as a bioactive therapeutic agent. It has been demonstrated that MgO_2_-incorporated biomaterials can achieve efficient local antibacterial treatment for implant-associated infections, and MgO_2_-based functional scaffolds can enable time-sequential controlled release of Mg^2+^ and reactive oxygen species to match the staged bone healing process [21, 22].

However, the translational potential of unmodified MgO_2_ is limited by a critical bottleneck: uncontrolled rapid degradation in proton-rich physiological environments. This induces a burst release of oxygen and hydrogen peroxide (H_2_O_2_), which does not align with the temporal window of periodontal tissue repair and may amplify local inflammatory cascades while damaging normal tissues [23]. Polydopamine (PDA) is a biomacromolecule with excellent biocompatibility and biodegradability, widely used for the surface modification and functionalization of materials. Its surface contains abundant catechol and amine groups that provide numerous binding sites [24]. Additionally, its surface groups can react with OH- and exert a buffering effect. Furthermore, PDA can attenuate excessive inflammatory cascades and provide an extracellular matrix-mimetic interface for cell adhesion due to its excellent biocompatibility [25, 26]. This modification transforms MgO_2_ from a simple oxygen donor into a multifunctional therapeutic component, enabling synergistic targeting of hypoxia and inflammation, the key pathological drivers of periodontitis, while supporting subsequent bone regeneration.

Accordingly, we constructed a PDA-coated magnesium peroxide (MgO_2_@PDA) nanoparticle-loaded Pluronic F127-sodium alginate (F127-SA) thermosensitive hydrogel delivery system. This hydrogel exists as a highly fluid sol at room temperature, facilitating easy injection into structurally complex periodontal pockets via a syringe. After injection into the periodontal pocket, the hydrogel is expected to undergo efficient gelation through a dual cross-linking mechanism: temperature-induced self-assembly of F127 chains forms an initial physical network, while ionic cross-linking between sodium alginate and divalent cations (endogenous Ca^2+^ from gingival crevicular fluid (GCF) and released Mg^2+^) may further stabilize the three-dimensional (3D) structure [27–30]. This hydrogel sustainably releases oxygen and magnesium ions at periodontitis lesions. Oxygen-mediated inhibition of anaerobic metabolism eliminates periodontal pathogens and ameliorates the local hypoxic microenvironment, while the bioactivity of magnesium ions modulates macrophage polarization, alleviates excessive inflammatory responses and promotes osteoblast migration, differentiation and mineralization. Together, these effects exert synergistic antibacterial, anti-inflammatory and osteogenic activities, providing a novel local delivery strategy for the comprehensive treatment of periodontitis.

## Methods

### Materials

Nano-sized magnesium oxide (MgO, purity ≥99.9%, spherical, 50 nm) was purchased from Macklin (Shanghai, China). H_2_O_2_ (30%, w/w aqueous solution) was obtained from China National Pharmaceutical Group Chemical Reagent Co., Ltd. (Shanghai, China). Dopamine hydrochloride (purity ≥98%) and sodium alginate (SA, viscosity 200–250 mPa·s) were purchased from Aladdin (Shanghai, China). Pluronic F-127 and Tris-HCl buffer (pH 8.5) were purchased from Beyotime (Shanghai, China). Lipopolysaccharide (LPS) from *Porphyromonas gingivalis* was obtained from Thermo Fisher (France). All other reagents and solvents were of analytical grade and used as received without further purification.

### Synthesis and characterization of MgO_2_@PDA

MgO_2_@PDA nanoparticles were synthesized via a two-step method. First, MgO_2_ nanoparticles were prepared by reacting MgO nanoparticles (1.2 g) with 30% H_2_O_2_ (20 mL) in anhydrous ethanol (20 mL) at room temperature for 4 h with continuous stirring [31]. The product was collected by centrifugation, washed with ethanol and freeze-dried. Subsequently, the as-synthesized MgO_2_ nanoparticles (50 mg) were dispersed in a dopamine hydrochloride solution (0.5 mg/mL in 10 mM Tris-HCl buffer, pH 8.5), sonicated for 15 min to obtain a uniform dispersion and stirred in the dark at room temperature for 4 h. The final MgO_2_@PDA nanoparticles were obtained by centrifugation, thorough washing with deionized water and ethanol and freeze-drying.

The microstructure was observed using a transmission electron microscope (TEM, X002F solaT IEF, USA) at 200 kV. The hydrodynamic particle size and zeta potential were measured with a laser particle size analyzer (Zetasizer Nano ZS90, UK). Phase structure was analyzed via X-ray diffraction (XRD, Bruker D8 Advance, Germany) with Cu-Kα1 radiation (λ = 1.5406 Å), a scanning range of 2θ = 10°–80° and a scanning speed of 5°/min. Fourier transform infrared (FTIR) spectra (4000–600 cm^−1^) were recorded using a Nicolet lS10 spectrometer (USA) with the KBr pellet method. Thermogravimetric analysis (TGA) was performed under a nitrogen atmosphere (Netzsch STA 449 F3 Jupiter^®^, Germany) at a heating rate of 10 K/min from 30°C to 600°C. Chemical composition was characterized by X-ray photoelectron spectroscopy (XPS, Thermo Escalab 250Xi, USA) with C 1 s, O 1 s, N 1 s and Mg 2p orbitals analyzed.

### Synthesis and characterization of the hydrogels

Pluronic F-127 (18%, w/v) and SA (1.5%, w/v) were dissolved in sterile PBS, incubated overnight at 4°C with gentle stirring to obtain a transparent F127-SA (FS) hydrogel precursor solution. Then, the MgO_2_@PDA nanoparticle suspension was added to the FS precursor solution and mixed uniformly to fabricate the MgO_2_@PDA/F127-SA (MPFS) hydrogel, with a final MgO_2_@PDA concentration of 1% (w/v). The pure FS hydrogel without nanoparticles served as the control group. In addition to the pure FS control hydrogel, hydrogels containing PDA nanoparticles alone (PDA group), Mg^2+^ alone (Mg^2+^ group) and uncoated MgO_2_ nanoparticles alone (MFS group) were prepared as control groups, with the concentration of each component matched to that in the experimental hydrogel. All solutions were prepared under sterile conditions for subsequent biological experiments.

Gelation kinetics at 37°C were quantified via the inverted vial assay. Briefly, 1 mL of hydrogel solution was added to a 5-mL glass vial and incubated in a water bath at 37°C. The time required for the solution to stop flowing upon inversion was recorded as the gelation time. Injectability was assessed by extruding the hydrogel precursor solution through a 25-gauge syringe needle at room temperature. Rheological properties were measured using a rotational rheometer (MCR 302, Anton Paar, Austria) equipped with a 25-mm parallel plate geometry and a fixed gap of 1.0 mm: (1) Thermosensitive gelation behavior was monitored via an oscillatory temperature ramp from 25°C to 40°C at a constant strain of 1.0% and frequency of 1 Hz; (2) Shear-thinning characteristics were evaluated through a continuous shear rate sweep spanning 0.01–100 s^−1^ at 25°C; (3) Self-recovery capability was determined by cyclic oscillatory tests with alternating low (0.1% strain, 120 s) and high (100% strain, 120 s) shear phases at 37°C. Attenuated total reflection (ATR)-FTIR spectra were recorded in the range of 4000–600 cm^−1^ using the same Nicolet iS10pectrometer. The microstructure of lyophilized hydrogel specimens was observed using a scanning electron microscope (SEM, TESCAN CLARA, Czech Republic) at an accelerating voltage of 5 kV.

#### Oxygen Release Quantification

An amount of 100 mg of 1% MFS and MPFS hydrogel was accurately weighed and submerged in 10 mL of release medium (PBS, pH 7.4 or pH 5.5) in 50 mL centrifuge tubes. Tubes were incubated in a thermostatic shaker (37°C, 100 rpm), and the dissolved oxygen (DO) concentration of the medium was tracked at predetermined time points (0, 1, 2, 4, 8, 12, 24, 48, 72, 96, 120, 144 and 168 h) via a calibrated DO meter (Mettler Toledo, SevenGo Pro, Switzerland).

#### Mg^2+^ Release Quantification

Samples were prepared as per the oxygen release protocol (100 mg hydrogel + 10 mL release medium, 37°C, 100 rpm). At the aforementioned time points, 1 mL of supernatant was harvested and an equal volume of pre-warmed fresh release medium was promptly supplemented to maintain constant volume. Supernatants were filtered through 0.22 μm sterile filters, and Mg^2+^ concentration was quantified via inductively coupled plasma optical emission spectrometry (ICP-OES, PerkinElmer Optima 8300, USA).

### *In vitro* antibacterial activity assays

Anaerobic co-culture systems were established in sterile tubes under anaerobic conditions (85% N_2_, 10% H_2_, 5% CO_2_). *Porphyromonas gingivalis* (ATCC 33277, *P. gingivalis*) and *Fusobacterium nucleatum* (ATCC 25586, *F. nucleatum*), the key periodontal anaerobic pathogens, were used in this study. The experimental groups were set as follows: (1) Bacterial control (bacterial suspension only); (2) FS hydrogel group (bacterial suspension + 100 mg FS hydrogel); (3) MFS hydrogel group (bacterial suspension + 100 mg MFS hydrogel); (4) MPFS hydrogel group (bacterial suspension + 100 mg MPFS hydrogel). All cultures were anaerobically incubated at 37°C for 48 h, with bacterial growth monitored via optical density at 600 nm (OD_600_) measurements at predefined time points. Post-incubation, the co-culture mixtures were serially diluted and each dilution was spread onto blood agar plates. The plates were then anaerobically incubated at 37°C, with outcomes documented by photography and bacterial colony counting. Bacteriostasis rate was calculated using formula (1):


(1) Bacteriostasis rate (%)=(RC-RT)RC×100%,

where R_C_ and R_T_ refer to the counting of control group and the experimental group.

### *In vitro* biofilm inhibition assays

#### Biofilm formation inhibition assay (crystal violet staining)

Sterile 12-well plates were prepared with groups per well: the experimental group contained pre-reduced medium, pre-sterilized hydrogel, logarithmic-phase bacterial suspension and 1% sucrose medium; the negative control group replaced the hydrogel with additional pre-reduced medium. After gentle mixing, plates were anaerobically incubated (85% N_2_, 10% H_2_, 5% CO_2_) at 37°C for 48 h. Following incubation, wells were stained with 0.1% crystal violet at room temperature for 20 min. Excess stain was rinsed thoroughly with deionized water, and wells were photographed. For quantification, 33% acetic acid was added to elute the stain; eluate was transferred to a 96-well plate, and absorbance at 590 nm was measured using a microplate reader.

#### Biofilm visualization via Confocal Laser Scanning Microscopy (CLSM)

Sterile confocal culture dishes were utilized for biofilm cultivation, with grouping and treatment conducted as described in Biofilm formation inhibition assay (crystal violet staining) section. The dishes were placed in an anaerobic atmosphere and statically incubated at 37°C for 48 h. After biofilm formation, samples were stained with the LIVE/DEAD bacterial staining kit (Beyotime, Shanghai, China) following the manufacturer’s protocols. Biofilm structures were then visualized via a confocal laser scanning microscope (Nikon AXR NSPARK, Japan).

### Biocompatibility

Hydrogel extract preparation: Hydrogel samples from each group were immersed in complete medium (DMEM for RAW264.7 macrophages and L929 fibroblasts; α-MEM for MC3T3-E1 pre-osteoblasts) at a material-to-medium ratio of 0.1 g/mL. The mixture was incubated at 37°C in a 5% CO_2_ atmosphere for 24 h or 72 h, then filtered through a 0.22-μm sterile filter to obtain extracts and used immediately within 24 h of preparation, without long-term storage.

### CCK-8 assay

#### Cell seeding and treatment

Cells were seeded in 96-well plates at 5 × 10³ cells per well, cultured for 24 h to allow attachment. Subsequently, 100 μL of hydrogel extract was added to each well (using pure complete medium as a control; MPFS hydrogel concentrations were 0.25, 0.5, 1, 2 and 4%). RAW264.7/L929 cells were incubated for 24, 48 and 72 h.

#### Viability assessment

At each time point, medium was removed, and 100 μL of 10% CCK-8 solution (Abbkine, Shanghai, China) was added. After 2 h incubation (37°C, 5% CO_2_), absorbance at 450 nm was measured (Thermo Multiskan FC, USA). Cell viability was calculated using formula (2):


(2) Cell viability (%)=ODs-ODbODc-ODb×100%,

where OD_s_, OD_c_ and OD_b_ refer to the absorbance of the experimental group, control group and 10% CCK-8 alone, respectively.

#### Hemolysis assay

Sheep blood anticoagulated with heparin was diluted at a 1:10 ratio with sterile normal saline to prepare a sheep red blood cell (SRBC) suspension. Aliquots of 0.2 mL SRBC suspension were added to 1.8 mL fresh hydrogel extract in sterile centrifuge tubes. Sterile normal saline and deionized water served as the negative and positive controls, respectively. All tubes were incubated in a shaking incubator (37°C, 100 rpm) for 1 h and then centrifuged at 3000 rpm for 15 min at room temperature. Tubes were photographed to record hemolysis, and the absorbance of the supernatants at 545 nm was measured using a microplate reader. Hemolysis rate was calculated using formula (3):


(3) Hemolysis rate (%)=ODs-ODnODp-ODn×100%,

where OD_s_, OD_n_ and OD_p_ denote the absorbance of the experimental group, negative control and positive control, respectively.

### Macrophage anti-inflammation assays

#### Quantitative real-time PCR (qRT-PCR) analysis of inflammatory cytokines

RAW264.7 macrophages were seeded in six-well plates at 2.0 × 10^5^ cells/well and incubated at 37°C with 5% CO_2_ for 24 h. Cells were divided into seven groups: control, LPS (1 μg/mL LPS) and LPS-treated groups supplemented with FS-, Mg^2+^-, PDA-, MFS- or MPFS-conditioned DMEM, respectively. After 24 h incubation, total RNA was extracted and the mRNA expression levels of *Il1b*, *Il6*, *Tnf* and *Inos* were determined via qRT-PCR, with *Actb* as the internal reference. The relative gene expression levels were calculated using the 2^−ΔΔCt^ method.

#### Macrophage polarization flow cytometry

RAW264.7 cells were seeded in 6-cm culture dishes at 5.0 × 10^5^ cells/dish and incubated until fully adherent. Cells were grouped and treated as described in qRT-PCR analysis of inflammatory cytokines section. Harvested cells (≈1.0 × 10^6^ cells/tube) were followed by the addition of fluorescently conjugated anti-CD86 (M1 marker) and anti-CD206 (M2 marker) antibodies. The tubes were incubated at 4°C in the dark for 30 min. Finally, cells were resuspended in fresh PBS solution and immediately analyzed via flow cytometry.

### Scratch wound healing assays

L929 mouse fibroblasts were routinely cultured in complete DMEM supplemented with 10% FBS (37°C, 5% CO_2_), then seeded in six-well plates (5 × 10^5^ cells/well) and cultured until 90–100% confluence. A sterile 200 μL pipette tip was used to create uniform scratches; cells were washed three times with sterile PBS to remove cellular debris. Immediately afterward, cells were treated with low-serum DMEM (1% FBS, negative control) or 1% FBS DMEM containing hydrogel extract (experimental groups supplemented with FS-, Mg^2+^-, PDA-, MFS- or MPFS-conditioned 1% FBS DMEM). Images were captured at 0, 12 and 24 h, and average scratch area was analyzed using ImageJ software. Migration rate was calculated using formula (4):


(4) Migration rate (%)=A0-ATA0×100%,

where A_0_ refer to the scratch area of 0 h, and A_T_ refer to the scratch area of 12 h or 24 h.

### MC3T3-E1 cell migration assay

MC3T3-E1 pre-osteoblast cells were resuspended in serum-free α-MEM at a density of 1 × 10^5^ cells/mL. A 200-μL aliquot of the cell suspension was added to the upper chamber of each Transwell insert, while 600 μL of complete α-MEM (control group) or the corresponding hydrogel-conditioned α-MEM (FS, Mg^2+^, PDA, MFS or MPFS group) was added to the lower chamber. After incubation at 37°C in a 5% CO_2_ atmosphere for 24 h, the non-migrated cells on the upper surface of the membrane were gently removed with a cotton swab. The migrated cells on the lower surface were fixed with 4% paraformaldehyde for 15 min and stained with 0.1% crystal violet solution for 20 min at room temperature. After rinsing thoroughly with deionized water and air-drying, the membranes were observed and photographed.

### Osteogenic differentiation evaluation assays

#### Alkaline phosphatase (ALP) staining and quantitative assay

MC3T3-E1 cells were seeded in 12-well plates and cultured until confluent. Medium was then replaced: the negative control used osteogenic induction medium, while the experimental group used osteogenic induction medium supplemented with sterile hydrogel-conditioned α-MEM (FS, Mg^2+^, PDA, MFS or MPFS group). Fresh medium and hydrogel-conditioned medium were refreshed every 3 days. On Day 7 of osteogenic induction, ALP staining was conducted via the BCIP/NBT method, with stained cells imaged under an inverted microscope. For ALP quantification, cell lysates were collected, and ALP activity was detected using a commercial ALP assay kit (microplate method). Total protein concentration in lysates was determined via a BCA protein assay to standardize ALP activity, with results expressed as ‘U/g protein’.

#### Alizarin red S (ARS) staining and mineralization quantification

Calcium nodule formation (mineralization) was evaluated via ARS staining on Day 21 of osteogenic induction. ARS staining was performed, and stained calcium nodules were imaged under an inverted light microscope. After imaging, stained, rinsed cells were air-dried at room temperature. Hexadecylpyridinium chloride solution was added to each well, and the plate was incubated on a shaker at room temperature. Absorbance was measured at 562 nm using a microplate reader to quantify calcium nodule content, with results expressed as ‘μg calcium/mg protein’.

#### Osteogenic marker gene and protein expression analysis

MC3T3-E1 cells were seeded in six-well plates, grouped into negative control and hydrogel experimental groups, and subjected to 7 days of osteogenic induction. For gene expression: total RNA was extracted via Trizol reagent, and qRT-PCR was performed to detect mRNA levels of osteogenic markers (*Runx2, Opn, Bglap* and *Col1a1*), with *Actb* as the internal reference. For protein expression: total cellular protein was extracted using RIPA lysis buffer (supplemented with protease/phosphatase inhibitors), protein concentration was determined via BCA assay, protein bands were visualized using an ECL imaging system and relative band gray values were quantified via ImageJ software.

#### Immunofluorescence staining of osteogenic markers

MC3T3-E1 cells were seeded in 12-well plates, grouped into negative control and hydrogel groups and subjected to osteogenic induction. Following induction, cells were fixed with 4% paraformaldehyde (room temperature, 15 min), permeabilized with 0.1% Triton X-100 (room temperature, 10 min) and blocked with 5% bovine serum albumin (room temperature, 1 h). Cells were incubated sequentially with anti-COL1A1/OPN primary antibody (4°C, overnight) and fluorescent secondary antibody (room temperature, 1 h, protected from light), then counterstained with DAPI (room temperature, 5 min, protected from light). Images were captured via an upright fluorescence microscope, and fluorescence intensity was quantified using ImageJ software.

### Animal experiments

All animal experimental protocols were approved by the Biomedical Ethics Committee of Nanjing Medical University (Approval No. 2401018). Specific pathogen-free (SPF) male Sprague-Dawley (SD) rats (150–200 g), provided by the Experimental Animal Center of Nanjing Medical University, were acclimated for 1 week under controlled conditions (22–25°C, 12 h light/dark cycle) with free access to standard laboratory chow and water. Rats were then randomly divided into four groups (*n* = 4 per group): healthy control, periodontitis model, FS hydrogel and MPFS hydrogel. The latter three groups underwent 2-week silk ligation to induce experimental periodontitis, and respective treatments were administered after ligature removal. The treatment groups received local injections of 20 μL of the corresponding hydrogel into the periodontal pocket of the maxillary second molar every 3 days for 4 weeks. The control group received an equal volume of sterile saline. Four weeks post-treatment, GCF samples were collected prior to euthanasia. The resulting bacterial suspension was transferred to a 12-well plate for quantitative biofilm formation analysis via crystal violet staining. Subsequently, rats were euthanized with an overdose of sodium pentobarbital. Maxillary alveolar bones were first scanned by micro-computed tomography (micro-CT) with parameters set as 90 kV, 88 μA and 50 μm pixel size, followed by 3D reconstruction using CTvox software. Thereafter, maxillary alveolar bone (for histological staining) and heart, liver, spleen, lung and kidney tissues (for Hematoxylin and eosin (HE) staining to evaluate biocompatibility) were harvested and fixed in 4% paraformaldehyde. Maxillary alveolar bone was continuously decalcified in decalcifying solution for 30 days, and all tissues were then paraffin-embedded, sectioned at 4 μm and stained with corresponding histological reagents per experimental requirements.

### Statistical analysis

All experimental data were statistically analyzed using GraphPad Prism 9 and Origin software. Results are presented as mean ± standard deviation (SD). Multiple group comparisons were conducted by one-way ANOVA followed by Tukey’s *post hoc* test, with significance levels denoted as *P < 0.05, **P < 0.01, ***P < 0.001 and ****P < 0.0001.

## Results

### Synthesis and characterization of MgO_2_@PDA nanoparticles

**Figure 1:**
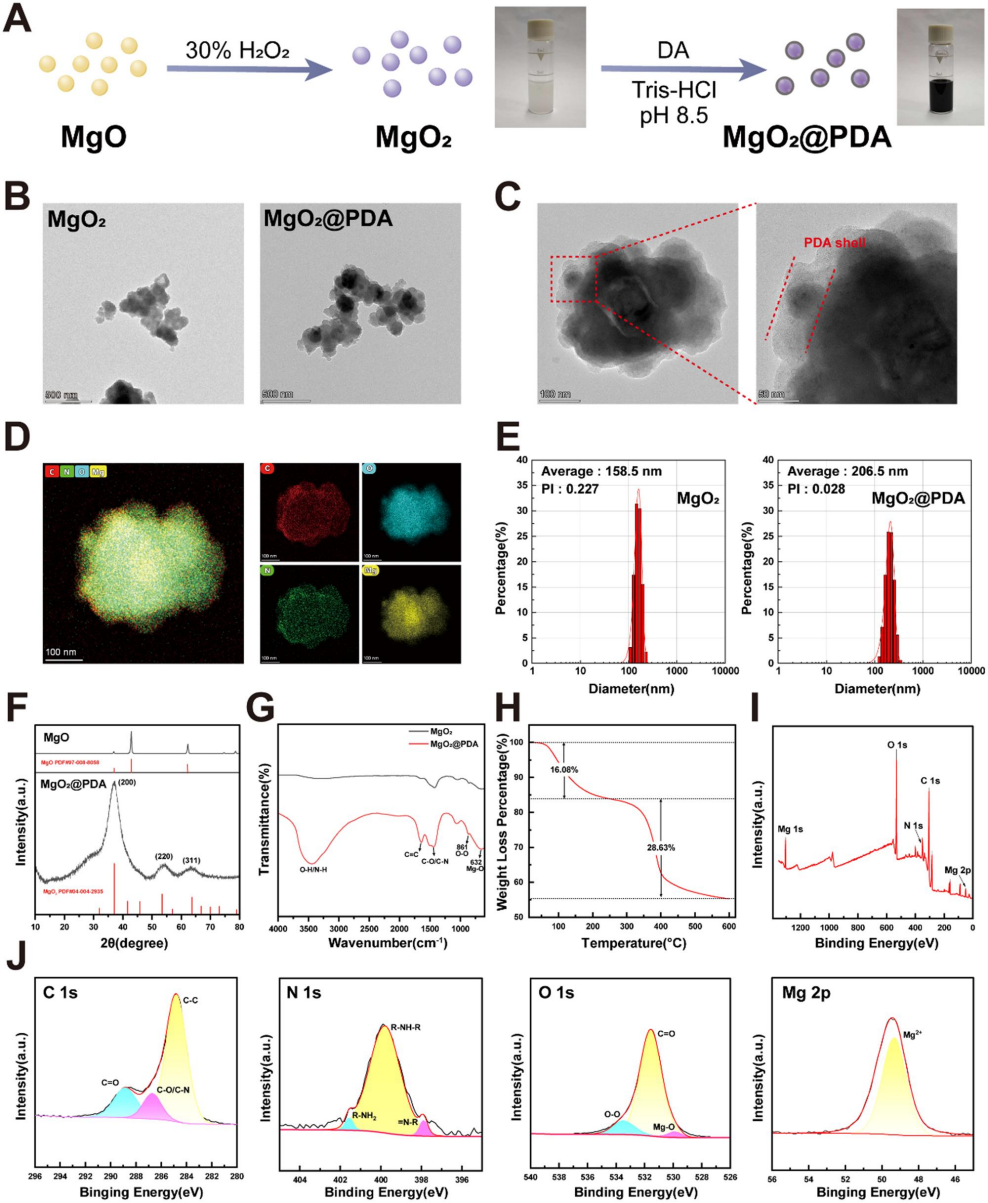
Synthesis and structural characterization of MgO_2_@PDA nanoparticles. (A) Schematic illustration of the synthetic route of MgO_2_@PDA nanoparticles. (B) TEM images of MgO_2_ nanoparticles (left) and MgO_2_@PDA nanoparticles (right). Scale bar: 500 nm. (C) High-magnification TEM image of MgO_2_@PDA nanoparticle, which reveals the core-shell structure with the PDA shell layer marked by red dashed lines. Scale bar: 100 nm and 50 nm. (D) EDS elemental mapping of MgO_2_@PDA (C, N, O, Mg). Scale bar: 100 nm. (E) Particle size distribution map of MgO_2_ (left) and MgO_2_@PDA (right). (F) XRD patterns of pristine MgO and MgO_2_@PDA. (G) FTIR spectra of MgO_2_ and MgO_2_@PDA. (H) TGA curve of MgO_2_@PDA nanoparticles recorded under nitrogen atmosphere. (I) Full survey XPS spectrum of MgO_2_@PDA nanoparticles. (J) High-resolution XPS spectra of the C 1s, N 1s, O 1s and Mg 2p for MgO_2_@PDA.

MgO_2_ nanoparticles were initially fabricated via the reaction of MgO nanoparticles with H_2_O_2_. Subsequently, a PDA coating was formed on the MgO_2_ surface through dopamine polymerization in a weakly alkaline solution, yielding MgO_2_@PDA nanocomposites (Figure 1A). The morphology and microstructure of the nanoparticles were characterized using TEM, and the results are shown in Figure 1B. The synthesized MgO_2_ nanoparticles exhibit a predominantly spherical to slightly irregular aggregate morphology, with individual primary particle sizes ranging from 100 to 180 nm, consistent with previously reported MgO_2_ nanoparticle morphologies synthesized via similar wet-chemical methods [32]. In contrast, the MgO_2_@PDA nanoparticles maintained a similar overall morphology but displayed distinct heterogeneous contrast, with the darker electron-dense regions corresponding to the MgO_2_ core and the lighter translucent regions corresponding to the PDA shell. High-magnification TEM images (Figure 1C) further confirmed the well-defined core-shell architecture of MgO_2_@PDA, revealing a continuous and uniform PDA shell layer enveloping the entire surface of the crystalline MgO_2_ nanocore. This uniform shell is critical for achieving consistent release kinetics of oxygen and Mg^2+^ ions [33].

To further verify the core-shell structure and elemental distribution of the nanocomposites, high-angle annular dark-field scanning transmission electron microscopy (HAADF-STEM) imaging (Figure S1) and energy-dis*p*ersive X-ray spectroscopy (EDS) mapping (Figure 1D) were conducted. The HAADF-STEM image exhibited clear Z-contrast, with the central region appearing significantly brighter than the peripheral region. This contrast difference arose from the higher atomic number of magnesium (*Z* = 12) compared to carbon (*Z* = 6) and nitrogen (*Z* = 7) in the PDA shell, providing direct visual evidence of the core-shell structure. Moreover, the EDS mapping confirmed the uniform co-distribution of Mg (from MgO_2_) and N (from PDA), further verifying the successful coating of the PDA shell onto the MgO_2_ surface.

The hydrodynamic size distribution and surface charge properties of the nanoparticles were evaluated using dynamic light scattering (DLS) and zeta potential measurements, respectively. DLS analysis (Figure 1E) showed that the average hydrodynamic diameter of the nanoparticles increased from 158.5 nm for unmodified MgO_2_ to 206.5 nm after PDA coating. The increase in hydrodynamic diameter was in good agreement with the PDA shell observed via TEM, considering the hydrated state of the nanoparticles in aqueous solution. Both samples exhibited narrow, near-normal particle size distribution curves and low polydispersity indices, indicating that the synthesized nanoparticles possess excellent uniformity and a well-controlled particle size distribution. Zeta potential measurements (Figure S2) showed that MgO_2_@PDA nanocomposites had a significantly higher absolute negative charge than unmodified MgO_2_ (****P < 0.0001), attributed to the abundant deprotonated catechol and hydroxyl groups on the PDA surface under physiological pH conditions [34]. The enhanced negative charge not only corroborates the successful deposition of the PDA layer but also induces strong electrostatic repulsion between adjacent nanoparticles, effectively inhibiting agglomeration.

The crystalline structure and phase purity of the nanoparticles were characterized using XRD analysis (Figure 1F). The XRD pattern of MgO_2_@PDA nanoparticles exhibited characteristic diffraction peaks corresponding to the (200), (220), (311) crystal planes at 2θ values of 37°, 54° and 64°, which closely match the characteristic peaks of the MgO_2_ standard card (PDF#04-004-2935), indicating the synthesis of high-purity MgO_2_; the absence of impurity peaks suggests that the PDA coating did not alter the core crystal structure of MgO_2_.

FTIR spectroscopy was employed to analyze the chemical composition and functional groups of the nanoparticles (Figure 1G): the MgO_2_ sample exhibited characteristic absorption peaks at 632 and 861 cm^−1^ corresponding to Mg-O and O-O bonds, respectively. The MgO_2_@PDA composite retained these peaks and exhibited additional PDA-specific features: a broad ∼3400 cm^−1^ peak (O-H/N-H stretching) and a ∼1610 cm^−1^ band (aromatic C=C stretching).

TGA under nitrogen (Figure 1H) revealed a 28.63% weight loss of MgO_2_@PDA at 200–600°C, close to pure MgO_2_’s theoretical decomposition loss (∼28.4%). This indicated overlapping thermal decomposition of PDA and oxygen-releasing decomposition of MgO_2_ in this temperature range.

XPS was used to characterize the surface chemical composition and bonding states of MgO_2_@PDA nanoparticles. In the full spectrum of MgO_2_@PDA (Figure 1I), characteristic peaks for C, N, O and Mg are clearly observable. The presence of the N 1 s characteristic signal provides direct elemental evidence for the successful coating of PDA, which is fully consistent with the results described above. Furthermore, after peak fitting of the high-resolution spectra for each element (Figure 1J), the C 1 s spectrum can be resolved into three characteristic peaks: C–C, C–O/C–N and C=O/C=N; the N 1 s spectrum can be fitted to three characteristic peaks: R–NH_2_, R–NH–R and R=N–R, corresponding to typical nitrogen-containing groups in the PDA structure; O 1 s peaks appear at 531.3 eV (C=O), 533.0 eV (–O–O–) and 530.0 eV (Mg-O); Mg 2p exhibits a symmetric peak at 49.45 eV (characteristic of Mg^2+^), further confirming the elemental composition and chemical bonding states of the MgO_2_@PDA nanoparticles.

Collectively, these results validate the successful fabrication of MgO_2_@PDA nanoparticles with favorable properties, supporting their further application in subsequent therapeutic studies.

### Characterization of MgO_2_@PDA/F127-SA composite hydrogels

**Figure 2:**
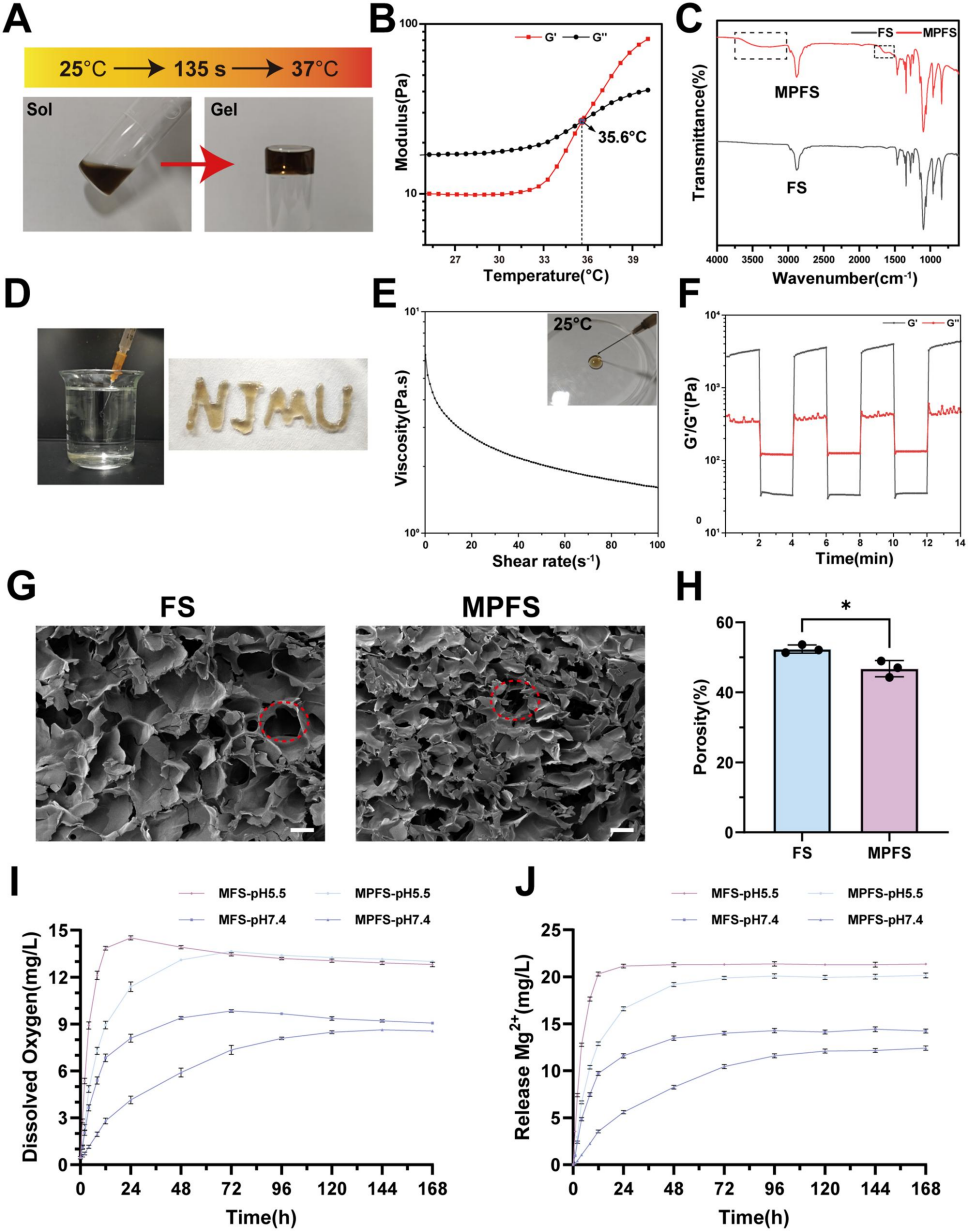
Physicochemical characterization, rheological properties and *in vitro* release behavior of MPFS thermosensitive hydrogels. (A) Photographs demonstrating the sol-gel transition of MPFS hydrogel: fluid sol state at 25°C, which transforms into a solid gel state at 37°C within 135 s. (B) Temperature-dependent rheological analysis of MPFS hydrogel, showing the crossover of G′ and G″ at 35.6°C. (C) FTIR spectra of pure FS and MPFS hydrogel. (D) *In vitro* injectability demonstration of MPFS hydrogel. (E) Viscosity-shear rate curve of MPFS hydrogel at 25°C. (F) Step-strain rheological test of MPFS hydrogel. (G) SEM images of FS (left) and MPFS (right) hydrogels (red dashed circles highlight pore structures; scale bar: 100 μm). (H) Quantitative analysis of hydrogel porosity from SEM images (data presented as mean ± SD, *n*=3; *P <0.05). (I) *In vitro* DO release profiles of MFS and MPFS hydrogels at pH 5.5 and pH 7.4 within 168 h. (J) *In vitro* Mg^2+^ release profiles of MFS and MPFS hydrogels at pH 5.5 and pH 7.4 within 168 h.

The thermosensitive gelation performance of the MPFS hydrogel was first evaluated via inverted vial test and rheological temperature-sweep analysis. As shown in Figure 2A, the MPFS hydrogel existed as a free-flowing sol at 25°C and rapidly transformed into a non-flowing solid gel within 135 s when heated to 37°C, demonstrating rapid and efficient thermal responsiveness. This macroscopic sol–gel transition was quantitatively verified by rheological temperature-sweep measurements (Figure 2B): the storage modulus (G′) and loss modulus (G″) crossed at 35.6°C, which was defined as the sol-gel transition temperature. This temperature is slightly below physiological body temperature, ensuring that the hydrogel can undergo rapid gelation after injection into periodontal pockets.

FTIR spectroscopy was employed to analyze the chemical structure and intermolecular interactions of the FS and MPFS hydrogels (Figure 2C). Compared with the FS hydrogel, the MPFS hydrogel exhibited a broadened absorption band at ∼3280 cm^−1^ (attributed to overlapping O–H/N–H stretching from PDA catechol/amine groups) and an enhanced weak peak at ∼1600 cm^−1^ (corresponding to aromatic C=C stretching in PDA benzene rings). No new impurity peaks or destruction of core functional groups were observed, verifying that the incorporation of MgO_2_@PDA did not alter the primary chemical structure of the hydrogel matrix, achieving excellent interfacial compatibility between the nanoparticles and hydrogel.

The injectability and mechanical stability of the MPFS hydrogel were further evaluated. As demonstrated in Figure 2D, the MPFS sol could be smoothly injected through a syringe needle into aqueous solution and maintained a stable gel state after gelation, with the ability to be molded into a defined shape, confirming its excellent injectability and shape retention. Steady-state flow rheological tests (Figure 2E) revealed that at 25°C, the hydrogel viscosity decreased drastically with increasing shear rate, exhibiting typical non-Newtonian shear-thinning behavior. This property ensures the hydrogel remains stable during storage, while thinning under the shear stress of injection, enabling facile delivery through fine needles. Step-strain cyclic tests (Figure 2F) further demonstrated that the hydrogel could rapidly and reversibly recover after alternating exposure to high and low strains, confirming its outstanding self-healing ability.

SEM was utilized to observe the 3D porous microarchitecture of the hydrogels. As shown in Figure 2G, FS hydrogel exhibited a highly porous, interconnected 3D network with large, irregular pore structures. In contrast, the MPFS hydrogel displayed a significantly denser, more compact network with smaller, more uniform pores. Quantitative porosity analysis (Figure 2H) further validated these morphological observations: the porosity of the MPFS hydrogel was significantly lower than that of the FS hydrogel (*P < 0.05). This densified microstructure not only enhances the mechanical stability of the hydrogel but also prolongs the retention time of nanoparticles at the lesion site [35], laying a foundation for sustained modulation of the periodontal pathological microenvironment.

The pH-responsive release profiles of O_2_ and Mg^2+^ from MFS and MPFS hydrogels were investigated at pH 5.5 and pH 7.4 (Figure 2I and J). Figure 2I depicts the DO release kinetics: both hydrogels exhibited an initial rapid release within 24 h followed by a gradual steady stage. Notably, the DO release level in the pH 5.5 group was significantly higher than that in the pH 7.4 group. This difference arises from the accelerated decomposition of MgO_2_ under acidic conditions, which promotes more efficient O_2_ generation. Figure 2J shows the Mg^2+^ release behavior, following a consistent pH-responsive trend. The Mg^2+^ release rate in the pH 5.5 group was markedly higher than that in the pH 7.4 group. In addition, MPFS showed more sustained and stable release behavior than MFS, demonstrating that the PDA shell effectively regulated the release kinetics.

### *In vitro* antibacterial activity evaluation of MPFS hydrogel

**Figure 3:**
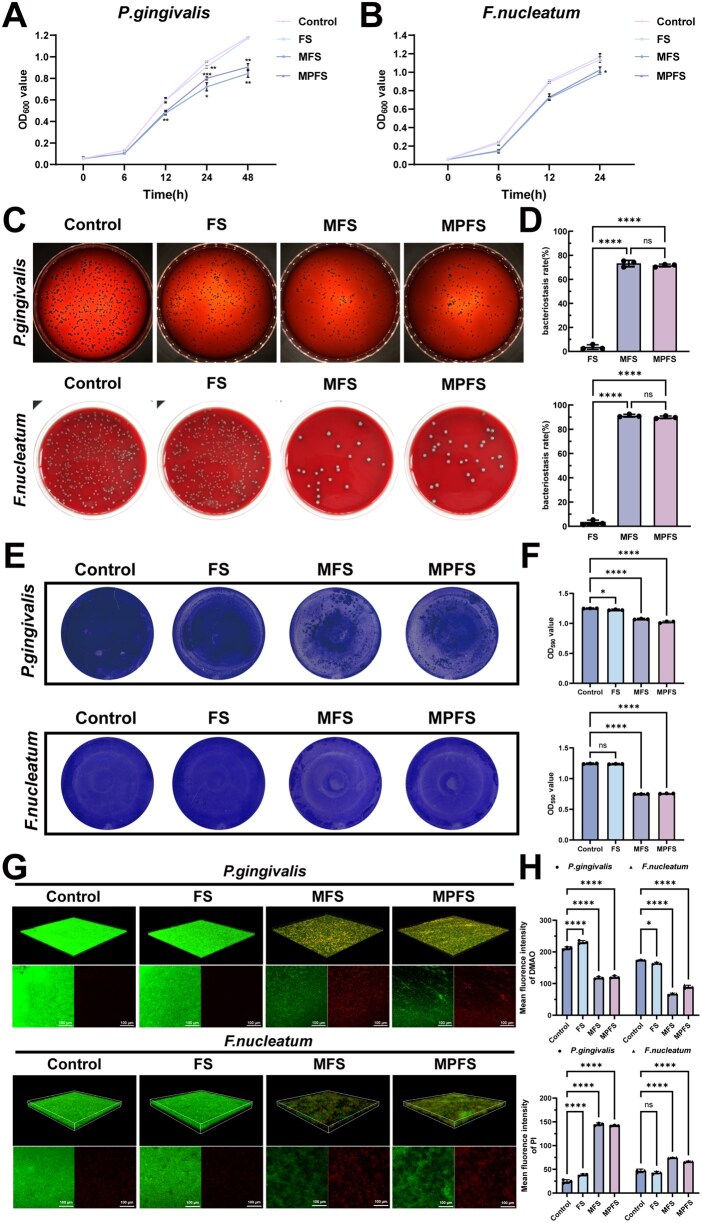
*In vitro* antibacterial activity of MPFS hydrogel against *P. gingivalis* and *F. nucleatum*. (A) Growth curves of *P. gingivalis* monitored by OD_600_ at 0, 6, 12, 24 and 48 h. (B) Growth curves of *F. nucleatum* monitored by OD_600_ at 0, 6, 12 and 24 h. (C) Representative colony images of *P. gingivalis* and *F. nucleatum*. (D) Quantitative analysis of bacteriostasis rates for *P. gingivalis* and *F. nucleatum*. (E) Crystal violet staining images of *P. gingivalis* and *F. nucleatum* biofilms. (F) Quantitative analysis of biofilm biomass for *P. gingivalis* and *F. nucleatum* measured by OD_590_. (G) CLSM images of live/dead stained *P. gingivalis* and *F. nucleatum* biofilms (green: DMAO-stained live bacteria; red: PI-stained dead bacteria; scale bar: 100 μm). (H) Quantitative analysis of mean fluorescence intensity of DMAO and PI for *P. gingivalis* and *F. nucleatum* biofilms. All data are mean ± SD, *n*=3, ns: no significance, *P <0.05, **P <0.01, ***P <0.001 and ****P <0.0001.

To assess the antibacterial potential of the MPFS hydrogel, two predominant periodontal pathogens—*P. gingivalis* and *F. nucleatum*—were employed. The hydrogel’s growth-inhibitory effect on bacterial proliferation over 48 h was evaluated by monitoring the OD_600_ in liquid culture medium. As illustrated in Figure 3A and 3B, the blank control and FS hydrogel groups exhibited typical sigmoidal growth curves for both pathogens. No significant difference in OD_600_ values was observed between the control and FS groups at testing time point, confirming that the blank F127-SA hydrogel matrix itself has no intrinsic antibacterial activity. In contrast, both MFS and MPFS hydrogels significantly suppressed bacterial proliferation starting from 12 h, when bacteria entered the logarithmic growth phase. These results demonstrate that the antibacterial activity of the composite hydrogel originates entirely from the MgO_2_@PDA nanocomposite component, and the PDA coating does not compromise the inherent antibacterial activity of MgO_2_.

To further quantify the antibacterial efficacy, we performed colony-forming unit (CFU) counting and calculated the antibacterial rate. Representative plate images (Figure 3C) showed that the MFS and MPFS groups exhibited a dramatic reduction in colony numbers for both *P. gingivalis* and *F. nucleatum*. Quantitative analysis of bacteriostasis rates (Figure 3D) revealed that the FS hydrogel had negligible antibacterial activity. In sharp contrast, both MFS and MPFS hydrogels exerted potent bactericidal effects, with bacteriostasis rates exceeding 70% against both pathogens.

Considering biofilm formation as a critical virulence factor of periodontal pathogens, the inhibitory capacity of the MPFS hydrogel against *P. gingivalis* and *F. nucleatum* biofilm formation was quantitatively assessed via crystal violet staining. As illustrated in Figure 3E, the blank control and FS hydrogel groups showed dense purple-stained biofilm layers covering well bottoms, indicating robust biofilm formation and adherence. In contrast, the MFS and MPFS hydrogel groups exhibited only faint staining, reflecting marked biofilm biomass reduction. For precise quantification, bound crystal violet was eluted, and OD_590_ was measured. Quantitative results (Figure 3F) fully corroborated visual observations: the MFS and MPFS groups had significantly reduced biofilm mass versus the blank control (****P < 0.0001), while the FS group had no significant impact on biofilm formation.

To investigate the effects of the MPFS hydrogel on the 3D structure of biofilms and the viability of bacteria, dual fluorescent staining was performed using DMAO (green fluorescence) and PI (red fluorescence), followed by observation via CLSM (Figure 3G). The control and FS groups formed a thick, structurally intact biofilm with intense green fluorescence, indicating a high density of viable bacteria. In contrast, the MFS and MPFS hydrogel-treated groups exhibited a significantly weakened green fluorescence signal, accompanied by a thinner and sparser biofilm structure and scattered red fluorescence foci. Quantitative analysis of mean fluorescence intensity (Figure 3H) revealed that the DMAO (live bacteria) intensity in the MFS and MPFS groups were reduced by approximately 50% for *P. gingivalis* and 60% for *F. nucleatum* compared to the control groups (****P < 0.0001), while the PI (dead bacteria) intensity increased significantly.

Collectively, these *in vitro* findings demonstrate that the MPFS hydrogel exerts robust antibacterial activity against both *P. gingivalis* and *F. nucleatum*, effectively inhibiting planktonic proliferation, colony formation and biofilm development.

### Evaluation of anti-inflammatory and pro-migration activity of MPFS hydrogels

Effective periodontal therapy requires simultaneous targeting of bacterial infection, excessive inflammation and impaired tissue repair. Herein, we systematically evaluated the *in vitro* biocompatibility, anti-inflammatory activity and pro-migratory potential of the MPFS hydrogel.

**Figure 4:**
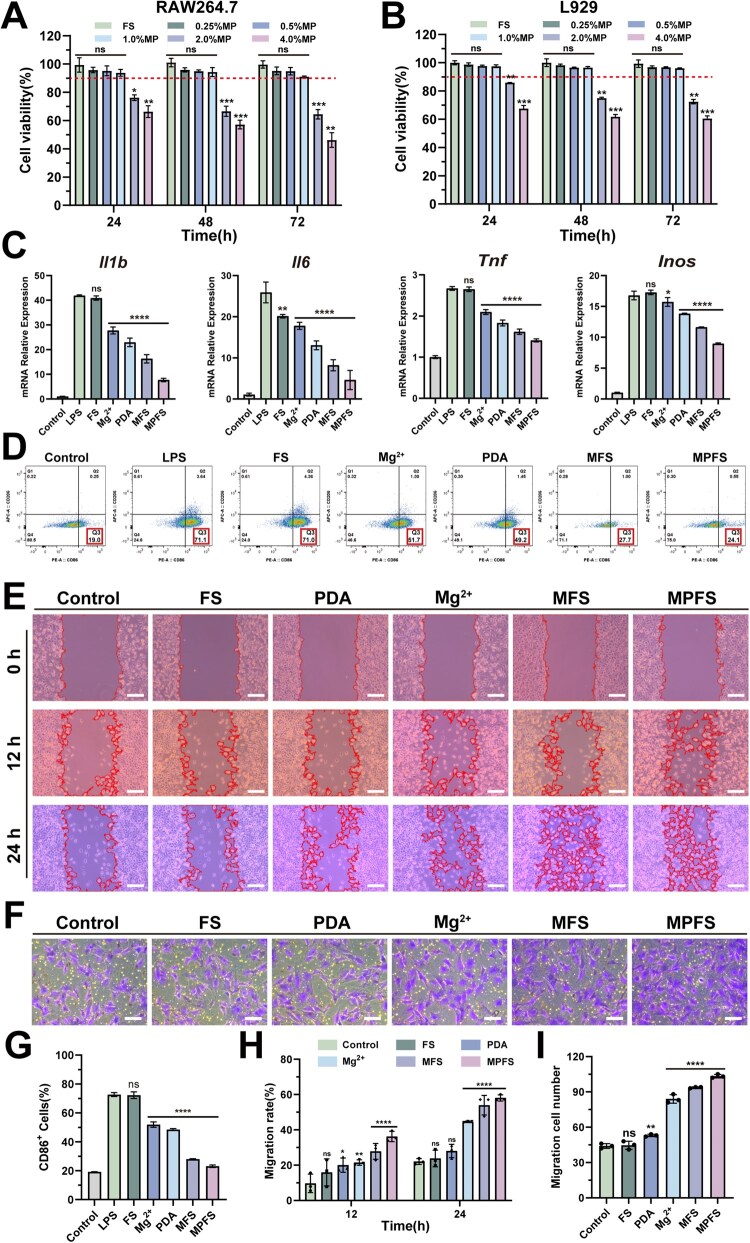
*In vitro* biocompatibility, immunomodulatory activity and pro-migratory effects of MPFS hydrogel. (A, B) Cell viability of RAW264.7 macrophages (A) and L929 fibroblasts (B) treated with different concentrations of MPFS hydrogel (0, 0.25, 0.5, 1.0, 2.0 and 4.0%) for 24, 48 and 72 h, determined by CCK-8 assay. The red dashed line indicates 90% cell viability. (C) Relative mRNA expression of *Il1b*, *Il6*, *Tnf* and *Inos* in RAW264.7 cells. (D) Flow cytometry analysis of RAW264.7 macrophage polarization (CD86^+^, M1; CD206^+^, M2). (E) Representative L929 fibroblast scratch wound images at 0, 12 and 24 h. Scale bar: 200 μm. (F) Representative Transwell migration assay images of MC3T3-E1 cells. Scale bar: 100 μm. (G) Quantitative analysis of CD86^+^ M1 macrophage ratio from flow cytometry data. (H) Quantitative analysis of L929 fibroblast migration rate at 12 and 24 h. (I) Quantitative analysis of migrated MC3T3-E1 cell number from Transwell migration assay. All data represent mean ± SD. *n*=3, ns: no significance, *P <0.05, **P <0.01, ***P <0.001 and ****P <0.0001.

To determine the safe working concentration of MPFS hydrogel for subsequent inflammation and migration assays, the CCK-8 assay was employed to evaluate the effect of MPFS hydrogel on the viability of RAW264.7 and L929 over a 72-h culture period. As shown in Figure 4A and 4B, at 1% concentration, the MPFS hydrogel maintained >90% viability of both cell types. In contrast, increasing the concentration to 2% significantly reduced the viability of both RAW264.7 and L929 cells. To further validate the biosafety of MPFS hydrogel for potential *in vivo* applications (e.g. local intra-pocket injection in periodontal treatment), a hemolysis assay was performed to assess its hemocompatibility, with representative results presented in Figure S3. The results showed that the hemolysis rate of MPFS hydrogel was <5% at concentrations ≤1.0%, indicating negligible hemolytic activity and excellent hemocompatibility. In contrast, the 2.0 and 4.0% concentration groups exhibited a hemolysis rate exceeding 5%, suggesting potential blood compatibility risks at these high concentrations. Therefore, a 1% concentration was used for subsequent experiments.

To assess the anti-inflammatory activity of the MPFS hydrogel, an *in vitro* inflammatory model was established via stimulation of RAW264.7 macrophages with LPS. qRT-PCR was then used to quantify the mRNA expression levels of key pro-inflammatory mediators (Figure 4C). LPS stimulation induced a dramatic upregulation of *Il1b*, *Il6*, *Tnf* and *Inos* compared to the untreated control group, confirming successful model establishment. Notably, treatment with MPFS hydrogel significantly downregulated the expression of all four pro-inflammatory genes (****P < 0.0001). Single-component control experiments revealed that both Mg^2+^ and PDA exhibited moderate anti-inflammatory activity, reducing pro-inflammatory gene expression by 20–35% and 30–40%, respectively, while the unmodified MFS hydrogel showed ∼50% reduction. The MPFS hydrogel achieved the most potent anti-inflammatory effect, demonstrating a clear synergistic effect between MgO_2_ and the PDA coating.

Flow cytometry analysis of CD86 expression (a specific marker for pro-inflammatory M1 macrophages) further confirmed these findings (Figure 4D and 4G). LPS stimulation increased the percentage of CD86^+^ M1 macrophages from 19 (control) to 71.1%. Consistent with the qRT-PCR results, Mg^2+^ and PDA single-component treatments partially reduced the proportion of CD86^+^ cells to 51.7 and 49.2%, respectively, while MFS reduced it to 27.7%. Most importantly, MPFS treatment significantly reduced the proportion of CD86^+^ cells to 24.1%, which was significantly lower than all single-component and unmodified MFS groups. Collectively, these results demonstrate that the MPFS hydrogel exerts potent anti-inflammatory activity by downregulating pro-inflammatory gene expression and inhibiting LPS-induced M1 macrophage polarization.

Fibroblast and osteoblast migration are critical early steps in periodontal tissue repair: fibroblasts synthesize extracellular matrix to regenerate connective tissue [36], while osteoblasts are responsible for alveolar bone regeneration [37]. We evaluated the pro-migratory activity of the MPFS hydrogel using scratch wound assays with L929 fibroblasts and Transwell migration assays with MC3T3-E1 preosteoblasts. As shown in Figure 4E and 4H, all groups showed limited scratch gaps healing at 12 h. After 24 h of culture, the control and FS hydrogel groups still exhibited limited scratch closure. Single-component experiments revealed that Mg^2+^ is the primary pro-migratory factor, increasing the migration area to 44.6%, while PDA showed only a minor effect. The unmodified MFS hydrogel further increased the migration area to 53.9%, and the MPFS hydrogel achieved the most potent pro-migratory effect, representing a 2.6-fold increase compared to the control group. Transwell migration assays using MC3T3-E1 preosteoblasts (Figure 4F and 4I) further confirmed the pro-regenerative potential of MPFS: the number of migrated MC3T3-E1 cells in the MPFS group was 2.34-fold higher than that in the control group (****P < 0.0001). The pro-migratory effect of MPFS is attributed to the sustained release of Mg^2+^ ions and O_2_, which have been shown to promote both fibroblast and osteoblast proliferation and migration [38, 39].

### *In vitro* assessment of the osteogenic activity of MPFS hydrogels

Alveolar bone resorption is the hallmark pathological change of periodontitis and the primary cause of tooth loss. Building on our previous findings of excellent biocompatibility and pro-migratory effects on MC3T3-E1 preosteoblasts, we systematically evaluated the pro-osteogenic capacity of the MPFS hydrogel at multiple levels.

**Figure 5:**
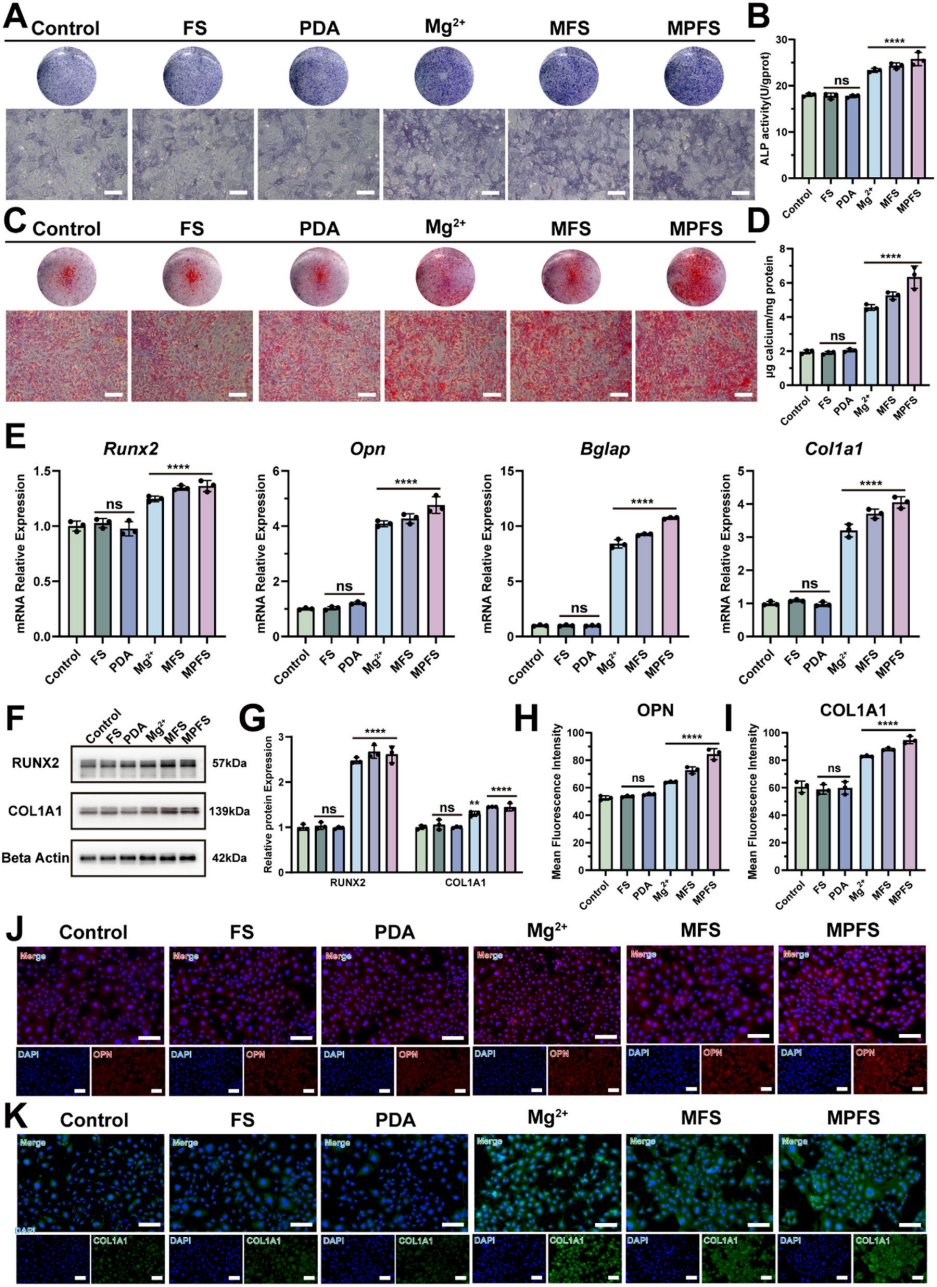
*In vitro* pro-osteogenic activity of the MPFS hydrogel in MC3T3-E1 preosteoblasts. (A) Representative ALP staining images of MC3T3-E1 cells after 7 days of osteogenic induction. Scale bar: 100 μm. (B) Quantitative analysis of ALP activity in MC3T3-E1 cells 7 days after osteogenesis induction. (C) Representative ARS staining images of mineralized nodules after 21 days of osteogenic induction. Scale bar: 100 μm. (D) Quantitative analysis of calcium content after 21 days of culture. (E) Relative mRNA expression levels of key osteogenic genes (*Runx2, Opn, Bglap* and *Col1a1*) measured by qRT-PCR. (F) WB analysis of osteogenic marker proteins (RUNX2, COL1A1) in MC3T3-E1 cells. (G) Quantitative analysis of the relative protein expression of RUNX2 and COL1A1, normalized to β-actin. (H) Mean fluorescence intensity of OPN from immunofluorescence staining. (I) Mean fluorescence intensity of COL1A1 from immunofluorescence staining. (J) Representative immunofluorescence images of OPN (red) in MC3T3-E1 cells. Scale bar: 100 μm. (K) Representative immunofluorescence images of COL1A1 (green) in MC3T3-E1 cells. Scale bar: 100 μm. All data represent mean ± SD. *n*=3, ns: no significance, *P <0.05, **P <0.01, ***P <0.001 and ****P <0.0001.

ALP activity is a well-recognized biomarker of early osteogenic differentiation. We performed ALP staining and quantitative activity assays after 7 days of osteogenic induction. As shown in Figure 5A, the MPFS hydrogel group exhibited the most intense blue-purple staining, indicating the highest ALP expression levels. Quantitative analysis (Figure 5B) revealed that the ALP activity in the MPFS group was 1.4-fold higher than that in the blank control group (****P < 0.0001). Single-component control experiments demonstrated that Mg^2+^ is the primary inducer of early osteogenic differentiation, while PDA alone showed no significant effect. To evaluate the effect of the MPFS hydrogel on late-stage osteogenic differentiation, we performed ARS staining and calcium content quantification after 21 days of osteogenic induction. As shown in Figure 5C, the MPFS group formed the densest and largest orange-red mineralized nodules, which were significantly more abundant than those in all control groups. Quantitative calcium content analysis (Figure 5D) confirmed these visual observations. Consistent with the ALP results, Mg^2+^ single-component treatment significantly increased calcium deposition, while PDA showed a non-significant effect. The superior osteogenic activity of MPFS compared to MFS may be attributed to the PDA coating-mediated sustained release of Mg^2+^ ions, which provides a continuous and stable stimulus for osteogenic differentiation throughout the entire differentiation process [40], rather than the transient high-concentration burst release observed with unmodified MgO_2_.

To further validate the pro-osteogenic properties of the MPFS hydrogel at the molecular level, qRT-PCR and WB analysis were performed to examine the expression of key osteogenic markers (*Runx2*, *Opn*, *Bglap* and *Col1a1*) in MC3T3-E1 cells following osteogenic induction. The qRT-PCR results (Figure 5E) revealed that compared to the blank control group, cells treated with the MPFS hydrogel exhibited significant upregulation of mRNA expression for all detected osteogenic genes (****P < 0.0001). At the protein level, WB analysis (Figure 5F and 5G) further confirmed the qPCR findings: compared to the blank control and FS hydrogel groups, the MPFS hydrogel group displayed markedly enhanced protein expression levels of RUNX2 and COL1A1. These results demonstrate that the MPFS hydrogel promotes osteogenic differentiation of MC3T3-E1 cells by upregulating the expression of key osteogenic markers at both the transcriptional and translational levels.

To further visualize the expression and distribution of osteogenic proteins, we performed immunofluorescence staining for OPN and COL1A1. To further visualize the expression and distribution of osteogenic proteins, we performed immunofluorescence staining for OPN and COL1A1. As shown in Figure 5J and 5H, OPN (red fluorescence) was diffusely distributed throughout the cytoplasm in the MPFS group, with a mean fluorescence intensity 1.6-fold higher than that in the control group (****P < 0.0001). Similarly, COL1A1 (green fluorescence) formed a dense fibrillar network in the MPFS group (Figure 5K and 5I), indicating active synthesis and secretion of type I collagen, with a mean fluorescence intensity 1.5-fold higher than that in the control group (****P < 0.0001). These results provide direct visual evidence that the MPFS hydrogel enhances the expression and correct localization of key osteogenic proteins, promoting extracellular matrix maturation and mineralization.

Collectively, these results demonstrate that the MPFS hydrogel exerts potent pro-osteogenic activity by promoting early osteogenic differentiation, enhancing late-stage matrix mineralization and upregulating the expression of key osteogenic markers at both the gene and protein levels.

### Evaluation of MPFS hydrogel for periodontitis treatment in an *in vivo* model

**Figure 6:**
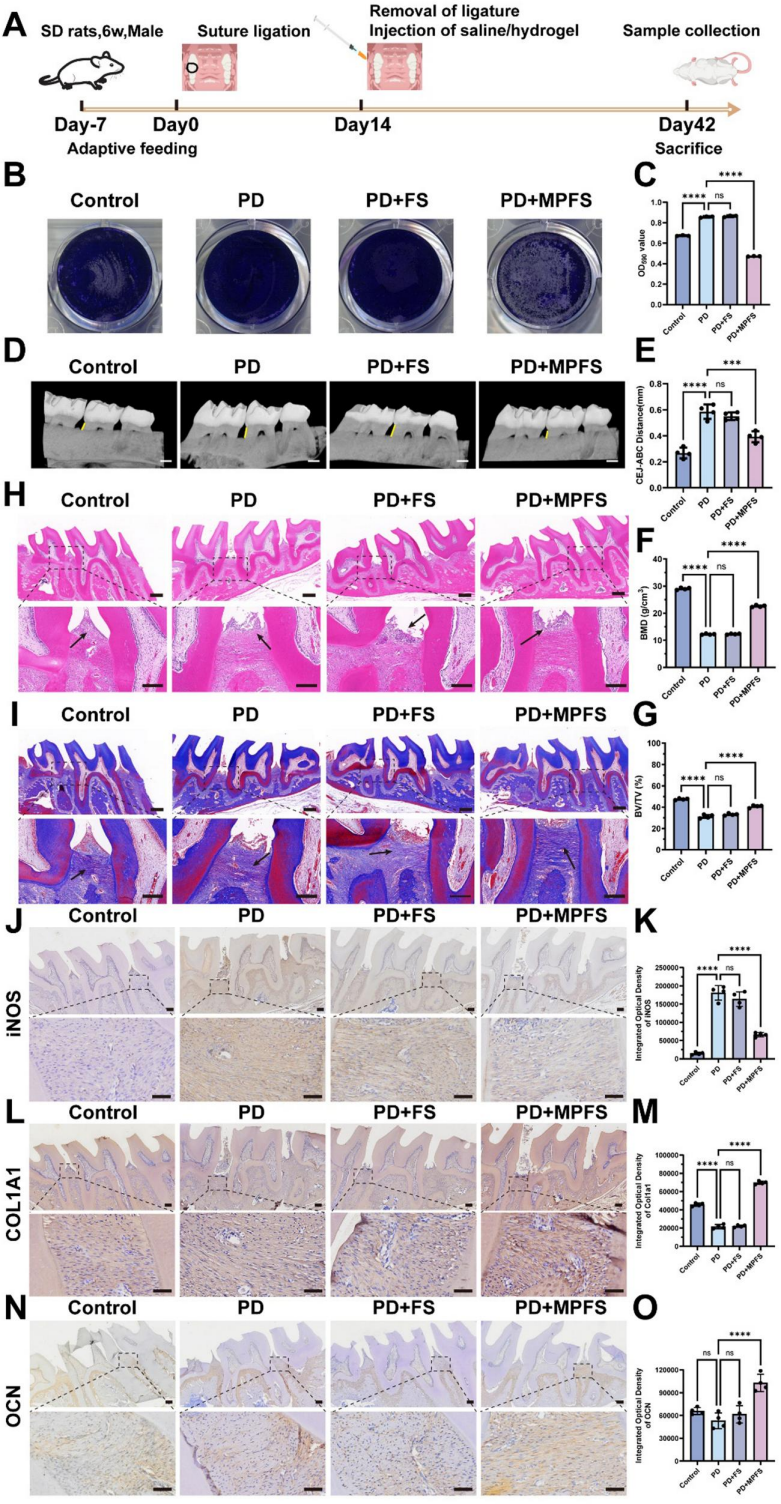
*In vivo* therapeutic efficacy of the MPFS hydrogel in a rat periodontitis model. (A) Schematic diagram of the animal experimental protocol. (B) Crystal violet staining images of bacterial cultures from rat GCF. (data presented as mean ± SD, *n*=3; ****P <0.0001) (C) Quantitative analysis of bacterial biomass from GCF cultures. (D) Micro-CT 3D reconstruction images of the maxillary molar region. Scale bar: 500 μm. (E-G) Quantitative analysis of CEJ-ABC distance (E), BMD (F) and BV/TV (G). (H) HE staining of periodontal tissue sections. Black arrows: the inflammatory cell infiltration area in the gingival epithelium and lamina propria. Scale bar: 500 μm and 200 μm. (I) Masson staining of periodontal tissue sections. Black arrows: the new collagen fiber and new bone matrix areas (blue-stained areas). Scale bar: 500 μm and 200 μm. (J, L and N) IHC staining of periodontal tissue sections for iNOS (J), COL1A1 (L) and OCN (N). Scale bar: 200 μm, 50 μm. (K, M, O) Quantitative analysis of IOD for iNOS (K), COL1A1 (M) and OCN (O) from IHC staining images. All data represent mean ± SD. *n*=4, ns: no significance, *P <0.05, **P <0.01, ***P <0.001 and ****P <0.0001.

To assess the *in vivo* therapeutic efficacy of the MPFS hydrogel, a ligature-induced periodontitis model was established in SD rats (Figure 6A). Experimental groups received local intra-pocket injections of saline, FS or MPFS hydrogel. After 4 weeks of treatment, maxillary bone specimens were harvested for comprehensive analyses of antibacterial activity, periodontal regeneration and biosafety.

Crystal violet staining was used to evaluate *in vivo* antibacterial efficacy. After 4 weeks, the biofilm biomass in the MPFS group was significantly lower than that in the periodontitis model (PD) and FS groups (****P < 0.0001) (Figure 6B and 6C), confirming the hydrogel’s potent *in vivo* antibacterial activity against periodontal pathogens.

High-resolution micro-CT scanning combined with 3D reconstruction was conducted to quantify alveolar bone loss (Figure 6D). Quantitative measurement of the cementoenamel junction-alveolar bone crest (CEJ-ABC) distance (Figure 6E) showed the PD group had a significantly greater distance than the healthy control (****P < 0.0001), confirming successful model establishment. Notably, the MPFS group exhibited a marked reduction in CEJ-ABC distance versus the PD and FS groups (***P < 0.001), indicating effective reversal of pathological alveolar bone loss. Analysis of bone microarchitectural parameters in defect areas (Figure 6F and 6G) further demonstrated that the MPFS group outperformed other model groups in bone mineral density (BMD) and bone volume fraction (BV/TV) (***P < 0.001), confirming enhanced bone regeneration.

HE staining was used to assess periodontal tissue morphology and inflammatory infiltration (Figure 6H). The healthy control group showed intact histological structures with minimal inflammatory cells, while the PD and FS groups exhibited severe pathological changes (dense inflammatory cell infiltration, widened periodontal ligament space and lost periodontal attachment). In contrast, the MPFS group had significantly reduced inflammatory infiltration.

Masson’s trichrome staining was conducted to evaluate collagen deposition (Figure 6I). The healthy control group had abundant, orderly blue collagen fiber bundles in the periodontal ligament, whereas the PD and FS groups showed disorganized, loosely arranged red fibrous tissue (indicating poor collagen maturation and fibrosis). The MPFS-treated defect area was filled with abundant, tightly arranged blue collagen fibers, resembling the healthy control group, confirming improved collagen synthesis and tissue remodeling.

Immunohistochemical (IHC) staining was performed to examine the expression of pro-inflammatory marker iNOS, bone matrix synthesis marker COL1A1 and bone mineralization marker OCN (Figure 6J, 6L and 6N). Dense iNOS-positive cells were observed in the PD and FS groups, while the MPFS group had significantly fewer iNOS-positive cells. For osteogenic markers, the MPFS group exhibited the strongest COL1A1 and OCN positive signals: continuous COL1A1 expression in newly formed bone indicated active matrix synthesis, and dense OCN signals colocalized with mineralized bone, signifying successful bone maturation. In contrast, the PD and FS groups had weak, diffuse signals. Statistical analysis (Figure 6K, 6M and 6O) confirmed that the MPFS group had significantly lower iNOS integrated optical density (IOD) values than the PD group (****P < 0.0001), and significantly higher COL1A1 and OCN IOD values than all other groups (****P < 0.0001).

Finally, histological analysis of major vital organs (Figure S4) showed normal morphological structures with no pathological abnormalities in the MPFS group, confirming the hydrogel’s favorable *in vivo* systemic biosafety.

## Discussion

Periodontitis represents a multifactorial inflammatory disease associated with several interconnected clinical challenges: pathogenic biofilms, sustained hyperinflammation, progressive alveolar bone destruction and hypoxic microenvironment. These overlapping barriers render monotherapies inadequate for positive outcomes, as effective treatment requires synchronized modulation of the pathological niche and active facilitation of tissue repair [41]. To address these challenges, we designed an injectable thermosensitive hydrogel system (MPFS). This multifunctional platform integrates stimuli‑responsive release, antibacterial activity, immunomodulation and pro‑osteogenic effects, enabling control of infection, resolution of inflammation and promotion of tissue repair.

Despite the promising antibacterial and osteogenic potential of MgO_2_-based nanoplatforms, unmodified MgO_2_ nanoparticles typically suffer from rapid, uncontrolled degradation, which can lead to local cytotoxicity, inconsistent therapeutic efficacy and impaired biocompatibility at effective antibacterial doses [42]. To address these limitations, we employed PDA as a multifunctional surface modifier to construct core–shell MgO_2_@PDA nanostructures. PDA coating has been demonstrated to reduce initial burst degradation and extend effective release duration [33]. The possible mechanism involves two aspects: the physical barrier effect of the PDA shell [43] and its pH-responsive degradation property [44]. Dopamine undergoes in-situ oxidative self-polymerization on the MgO_2_ surface to form a dense polymer shell, which may act as a physical diffusion barrier to reduce water molecule access to the MgO_2_ core and slow its hydrolysis, while also limiting the diffusion of hydrolysis products to inhibit burst release of active components. Notably, the PDA shell exhibits inherent pH-responsive degradation characteristics: it maintains high stability under neutral physiological conditions to ensure sustained slow release, but may become loose in the acidic inflammatory microenvironment due to weakened hydrogen bonds and π–π stacking interactions, moderately accelerating MgO_2_ hydrolysis to achieve microenvironment-responsive release of Mg^2+^ and oxygen. In addition to stabilizing MgO_2_, the PDA shell provides favorable biocompatibility, mild antioxidant activity and enhanced cell-material interactions, while preserving the oxygen-generating and Mg^2+^-releasing bioactivity of the inner core. This design thereby endows MgO_2_ nanoparticles with improved stability and biosafety while preserving their core therapeutic activities, making them more suitable for integration into a hydrogel system for periodontal therapy.

The MgO_2_@PDA nanocomposite serves as the bioactive core of the MPFS hydrogel, providing multimodal therapeutic functions. PDA coating enhances the colloidal stability and biocompatibility of MgO_2_ nanoparticles [45], while also exerting intrinsic anti‑inflammatory effects through reactive oxygen species scavenging and NF‑κB signaling suppression [46]. Meanwhile, MgO_2_ acts as a sustained oxygen generator that undergoes controlled acid-triggered decomposition to continuously release O_2_ in the periodontal microenvironment [18]. Since key periodontal pathogens such as *P. gingivalis* are obligate anaerobes that thrive at oxygen tensions below 5% [47], the elevated local oxygen level may contribute to the antibacterial effect by relieving hypoxia and suppressing their proliferation and virulence. By combining anti‑inflammatory, antibacterial and oxygen‑releasing capacities, MgO_2_@PDA synergistically remodels the pathological periodontal niche, creating a favorable milieu for subsequent tissue regeneration. Notably, the MPFS hydrogel acts on multiple components of the pathological cascade by combining antibacterial effects with immunomodulatory activity. Periodontitis progression is driven by a self‑reinforcing loop: anaerobic bacterial colonization triggers excessive inflammation, which further reduces local oxygen tension and promotes bacterial persistence [48]. Our *in vitro* tests confirmed MPFS hydrogel directly inhibited *P. gingivalis* and *F. nucleatum* proliferation and disrupted biofilm formation. Mechanistically, elevated oxygen tension may suppress bacterial pyruvate-ferredoxin oxidoreductase activity, thereby impairing anaerobic energy metabolism and compromising bacterial survival, without triggering rapid bacteriolysis or excessive endotoxin release [49]. Furthermore, MPFS treatment downregulated the expression of IL‑1β, IL‑6 and TNF‑α and reduced the proportion of CD86^+^ M1 macrophages. This dual action enables efficient infection control and inflammation resolution, which are prerequisites for successful periodontal regeneration. Persistent inflammation and hypoxia severely compromise osteogenesis by inhibiting osteoblast proliferation and differentiation while enhancing osteoclast activity, greatly limiting the performance of conventional osteogenic biomaterials [50]. In this study, the MPFS hydrogel reduced local inflammation and relieved hypoxic stress, thereby creating a more favorable milieu for osteoblastic function. In addition to these environmental improvements, the pro-osteogenic effects of MPFS may be linked to two supportive mechanisms reported in the literature. First, controlled Mg^2+^ release has been widely associated with enhanced osteogenic differentiation, which has been attributed to the activation of signaling pathways such as PI3K/Akt in preosteoblasts [51]. Second, the hydrophilic and porous structure of the alginate-containing hydrogel provides a favorable physical scaffold for cell adhesion, spreading and extracellular matrix deposition, which indirectly supports osteogenic maturation and mineralization [52, 53].Notably, magnesium-containing materials have been widely shown to promote ALP activity and matrix mineralization in preosteoblasts, in agreement with our present findings that MPFS significantly enhanced ALP expression, calcified nodule formation and the expression of osteogenic factors.

Of particular significance, the favorable osteo-regenerative potential identified *in vitro* was found to translate effectively to functional alveolar bone restoration in a rat model of periodontitis. Treatment with the MPFS hydrogel led to notable improvements in structural and morphometric bone parameters, as evidenced by micro‑CT evaluation, along with enhanced expression of COL1A1 and OCN within periodontal lesions. These outcomes are consistent with growing evidence suggesting that osteoimmunomodulatory biomaterials capable of concurrently modulating inflammation and supporting osteogenic differentiation yield superior bone regeneration compared with traditional biologically inert scaffolds [54]. The MPFS hydrogel integrates antimicrobial, inflammatory and pro-osteogenic activities in a single system. In this way, it promotes a regenerative tissue response rather than merely targeting isolated aspects of periodontal pathogenesis. Together, these *in vivo* results demonstrate that the MPFS hydrogel may exert combined therapeutic effects to support improved periodontal tissue repair and regeneration.

In summary, the MPFS hydrogel integrates multiple functionalities into a single platform, including injectable thermosensitive gelation, sustained release of oxygen and Mg^2+^, targeted antibacterial activity, anti-inflammatory effects and pro-osteogenic capacity. By simultaneously addressing the core pathological features of periodontitis—anaerobic bacterial infection, excessive inflammation and impaired bone regeneration—this system represents a promising candidate for localized periodontal therapy. Nevertheless, several notable limitations warrant further investigation. Specifically, direct measurements of key physicochemical parameters—such as pH evolution, H_2_O_2_ and ROS levels in release media and cell extracts—were not performed. These factors may modulate the hydrogel’s antibacterial activity, macrophage polarization responses and cytotoxicity in a concentration-dependent manner. Moreover, the detailed molecular mechanisms underlying its therapeutic effects remain to be elucidated. To address these gaps, future studies will first conduct comprehensive characterization of the dynamic changes in these physicochemical factors and subsequently employ transcriptomic and proteomic approaches to identify the key signaling pathways mediating its antibacterial, anti-inflammatory and osteogenic effects. Together, these investigations will provide a complete mechanistic understanding of the therapeutic action of the MPFS hydrogel.

## Conclusion

In this study, we developed a magnesium peroxide-based thermosensitive injectable hydrogel (MPFS) to address the multifactorial pathological features of periodontitis. The MPFS hydrogel demonstrated mild and sustained release of oxygen and magnesium ions, conferring the ability to inhibit anaerobic bacterial proliferation, attenuate inflammatory responses and exert potential osteogenic effects. *In vivo* studies demonstrated that local injection of MPFS into periodontal pockets significantly attenuated periodontal inflammation and ameliorated alveolar bone loss. Additionally, the MPFS hydrogel exhibited favorable biocompatibility in both *in vitro* and *in vivo* evaluations. Collectively, these findings suggest that the MPFS hydrogel represents a potential strategy for the minimally invasive local treatment of periodontitis.

## Supplementary Material

rbag098_Supplementary_Data

## Data Availability

Data will be made available on request.
