# Supplementary material for: Multifunctional thermosensitive hydrogel for synergistic antibacterial, anti-inflammatory and osteogenic effects to promote periodontal regeneration in periodontitis treatment
Source: Regen Biomater. 2026 May 21;13:rbag098. doi: 10.1093/rb/rbag098 (PMC13264424; doi:10.1093/rb/rbag098)
Supplement: rbag098_Supplementary_Data [file rbag098_supplementary_data.docx]

**Multifunctional Thermosensitive Hydrogel for Synergistic Antibacterial, Anti-Inflammatory and Osteogenic Effects to Promote Periodontal Regeneration in Periodontitis Treatment**

Long Wang^1,2,3^, Mingxi Wang^1,2,3^, Runzi Zhang^1,2,3^, Wanmeng Wang^1,2,3^, Jin Wu^1,2,3*^, Chunbo Tang^1,2,3*^

1 Department of Dental Implantology, The Affiliated Stomatological Hospital of Nanjing Medical University, Nanjing 210029, China

2 State Key Laboratory Cultivation Base of Research, Prevention and Treatment for Oral Diseases (Nanjing Medical University), Nanjing 210029, China

3 Jiangsu Province Engineering Research Center of Stomatological Translational Medicine (Nanjing Medical University), Nanjing 210029, China

**Correspondence:**

[cbtang@njmu.edu.cn](mailto:cbtang@njmu.edu.cn) (Chunbo Tang)

[wujin@stu.njmu.edu.cn](mailto:wujin@stu.njmu.edu.cn) (Jin Wu)

**
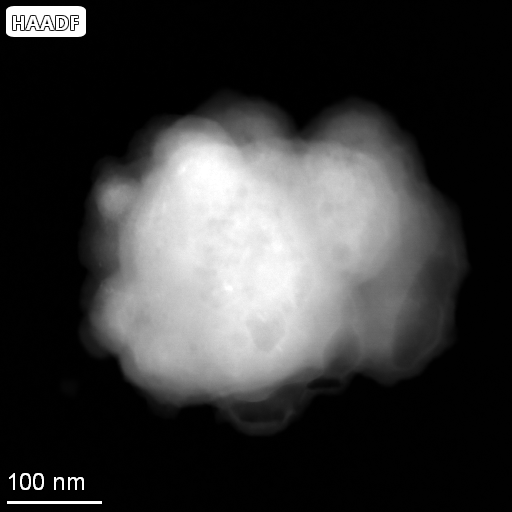
**

**Figure S1.** HAADF image of MgO₂@PDA. Scale bar: 100 nm.

**
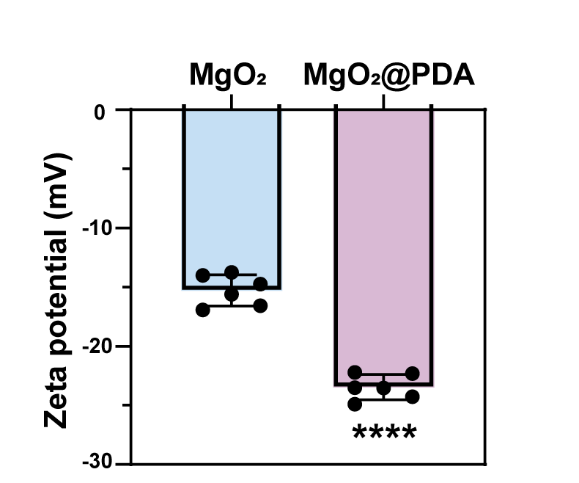
**

**Figure S2.** Zeta potential of MgO₂ and MgO₂@PDA (data presented as mean ± SD, n=6, **** *P*<0.0001).


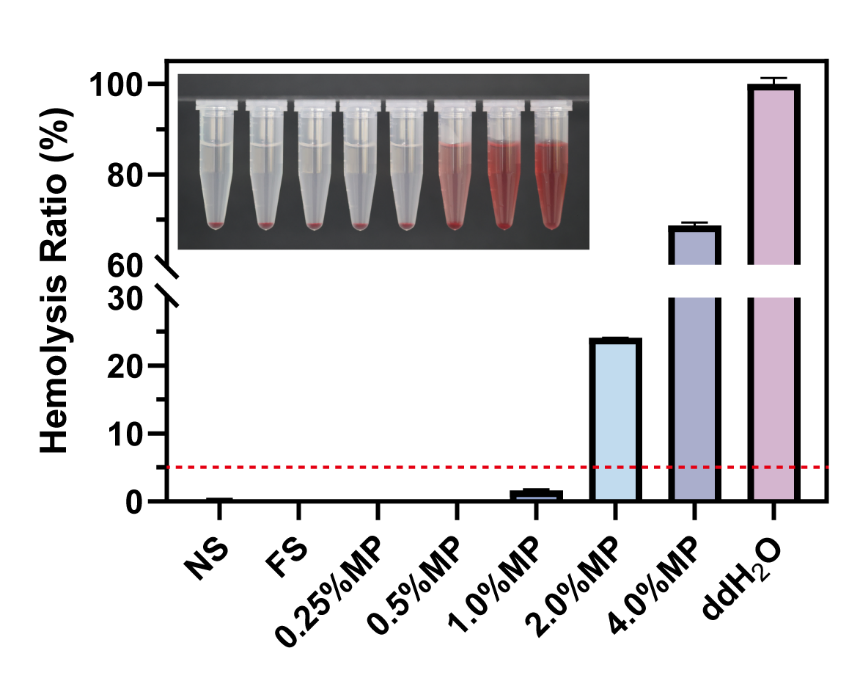


**Figure S3.** Representative images of hemolysis assay and quantitative analysis of hemolysis rate. The red dotted line indicates a hemolysis rate of 5%.


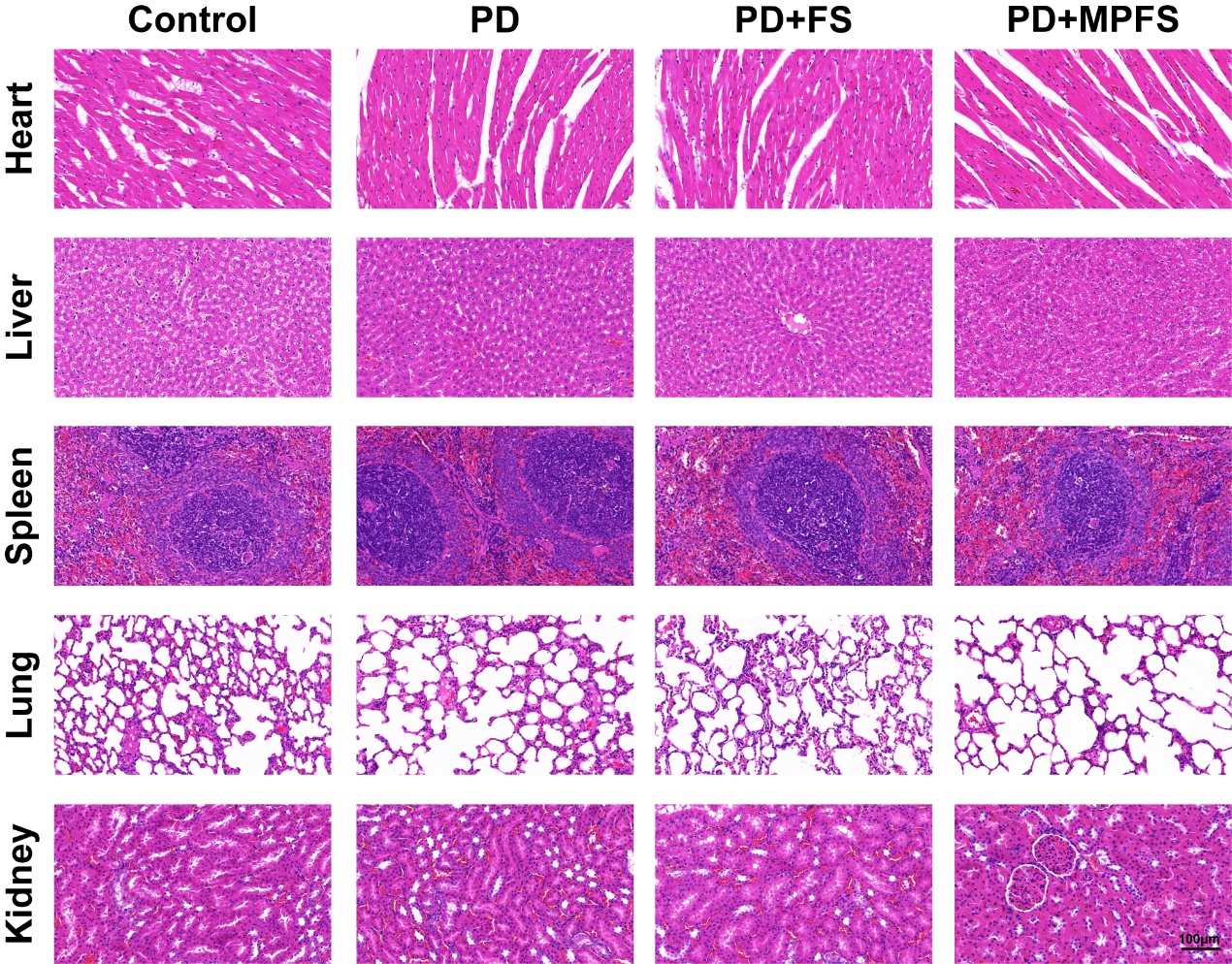


**Figure S4.** HE-stained images of major rat organs (heart, liver, spleen, lung, kidney) after 4 weeks of treatment.

**Table 1.** qRT-PCR primers Sequence

| Primers | Oligo sequences 5’-3’ |
| --- | --- |
| *Actb*-F | GTGACGTTGACATCCGTAAAGA |
| *Actb*-R | GCCGGACTCATCGTACTCC |
| *Il1b*-F | GAAATGCCACCTTTTGACAGTG |
| *Il1b*-R | TGGATGCTCTCATCAGGACAG |
| *Il6*-F | TAGTCCTTCCTACCCCAATTTCC |
| *Il6*-R | TTGGTCCTTAGCCACTCCTTC |
| *Tnf*-F | CCTGTAGCCCACGTCGTAG |
| *Tnf*-R | GGGAGTAGACAAGGTACAACCC |
| *Inos*-F | GGAGTGACGGCAAACATGACT |
| *Inos*-R | TCGATGCACAACTGGGTGAAC |
| *Runx2*-F | GCCCAGGCGTATTTCAGATG |
| *Runx2*-R | GGTAAAGGTGGCTGGGTAGT |
| *Opn*-F | TCCAATCGTCCCTACAGTCG |
| *Opn*-R | GGGACTCCTTAGACTCACCG |
| *Bglap*-F | GCAATAAGGTAGTGAACAGACTCC |
| *Bglap*-R | CCATAGATGCGTTTGTAGGCGG |
| *Col1a1*-F | CCACCCCAGCCGCAAAGAGTC |
| *Col1a1*-R | GTCATCGCACACAGCCGTGC |
